# Adherence to, and Persistence of, Antidepressant Therapy in Patients with Major Depressive Disorder: Results from a Population-based Study in Italy

**DOI:** 10.2174/1570159X20666220411092813

**Published:** 2023-03-08

**Authors:** Marco Di Nicola, Bernardo Dell’Osso, Ilaria Peduto, Riccardo Cipelli, Anna Cristina Pugliese, Maria Salvina Signorelli, Antonio Ventriglio, Giovanni Martinotti

**Affiliations:** 1 Department of Psychiatry, Fondazione Policlinico Universitario Agostino Gemelli IRCCS, Rome, Italy; Department of Neuroscience, Section of Psychiatry, Università Cattolica del Sacro Cuore, Rome, Italy;; 2 Department of Biomedical and Clinical Sciences Luigi Sacco, Aldo Ravelli Center for Neurotechnology and Brain Therapeutic, University of Milan, Milan, Italy;; 3 IQVIA Solutions Srl, Milano, Italy;; 4 Medical Department Lundbeck, Italia;; 5 Department of Clinical and Experimental Medicine, Psychiatry Unit, University of Catania, via Santa Sofia 78, 95123 Catania, Italy;; 6 Department of Clinical and Experimental Medicine, University of Foggia, Foggia, Italy;; 7 Department of Neuroscience, Imaging and Clinical Sciences, University “G. D'Annunzio” of Chieti - Pescara, Chieti, Italy;; 8 Psychopharmacology, Drug Misuse & Novel Psychoactive Substances Research Unit, School of Life and Medical Sciences, University of Hertfordshire, Hertfordshire, UK;; 9 SRP “Villa Maria Pia”, Rome, Italy

**Keywords:** Adherence, antidepressants, major depressive disorders, persistence, retrospective study, real-world evidence

## Abstract

**Background:**

Major depressive disorders represent a significant burden to society, and it is recommended that antidepressant therapy should last at least 6 months. In Italy, antidepressant use in clinical practice was reported to increase by 1.7% in 2020 compared to 2019, but only 40% of new prescriptions are characterized by a treatment duration longer than 3 months.

**Objective:**

The study aims to describe adherence and persistence to therapy in a subset of antidepressants (citalopram, duloxetine, escitalopram, paroxetine, sertraline, venlafaxine) vs. vortioxetine in Italy during a 2-year period from 2017 to 2019.

**Methods:**

A retrospective analysis of the longitudinal patient database reporting data from general practitioners on drug prescriptions in Italy was carried out in a cohort of 8,235 adult patients who were prescribed antidepressants.

**Results:**

Overall, 32.4% of the patients adhered to treatment for ≥6 months over a 1-year period. Vortioxetine had a lower risk of low adherence compared to duloxetine, paroxetine, and venlafaxine and a higher risk compared to citalopram, escitalopram, and sertraline. 68.7% of patients discontinued treatment during follow-up. The greatest percentage of patients continuing therapy was seen with duloxetine, while citalopram was associated with the highest proportion of patients discontinuing therapy. No significant differences in discontinuation were observed when comparing vortioxetine to the other antidepressants.

**Conclusion:**

Adherence results were considerably less than the 6-month recommendation in this real-world analysis of antidepressant therapies. Also, persistence to therapy was low, with most patients discontinuing treatment. Thus, there is a need for interventions to help patients adhere to their planned therapy.

## 1 INTRODUCTION

In Italy, a number of studies have provided estimates of the prevalence of major depressive disorders (MDDs). In a nationwide study carried out in 2002 by the Italian Society of General Medicine (SIMG) involving 191 primary care physicians, the prevalence of MDDs ranged from 7.8% to 9.0% in different geographic areas, with an overall prevalence of 8.4% [1]. Depression was also associated with severe physical illness and disability. In a later community survey from 2010 on nearly 5,000 individuals in Italy, the lifetime prevalence of MDDs, according to the Diagnostic and Statistical Manual of Mental Disorders, Fourth Edition (DSM-IV) criteria, was 4.3% in males and 11.5% in females [2]. These results are similar to the results of a study by the European Study of the Epidemiology of Mental Disorders (ESEMeD), which reported a lifetime prevalence of MDDs of 10.1% in Italy [3]. Overall, this indicates that the prevalence of MDDs is high in the general population and that MDDs are associated with significant societal impact as it does not only lead to impaired functioning and increased risk of suicide and morbidity but it results in a substantial economic burden and increased healthcare costs [4, 5]. In fact, globally, depression is a leading cause of disability [6].

General practitioners (GPs) play a key role in the management of depression, and antidepressants are currently the main class of drugs used to treat MDDs [7]. In the above-mentioned study by Carta *et al.*, for instance, in general practice in Italy, 4.7% of all subjects were currently on antidepressants [2]. Moreover, in a national report from 2020 by AIFA in Italy, antidepressants represented 3.7% of drug consumption by the national health service, with an increase of 1.7% compared to 2019 [8]. Notwithstanding their undisputedly common use, however, the use of antidepressants remains an area of controversy in terms of diagnosis, efficacy, safety, and choice of antidepressants [9, 10].

In MDDs, poor adherence, non-persistence, and early interruption of pharmacological treatment are associated with poorer clinical outcomes and increased costs of care [11]. As with any medical treatment, adherence to prescribed therapy is a crucial aspect of treatment. The World Health Organization (WHO) defines adherence to therapy as “the extent to which a person’s behaviour – taking medication, following a diet, and/or executing lifestyle changes, corresponds with agreed recommendations from a health care provider” [12]. Non-adherence includes “failing to fill prescriptions, delaying prescription fills, reducing the strength of the dose taken, and reducing the frequency of administration. It can also include the failure to keep appointments or to follow recommended lifestyle or dietary changes” [13].

For antidepressants, adherence to therapy is essential in achieving successful outcomes [14]. Despite the recommendation for depression management reported in the NICE clinical guidelines 2009 suggests continuing antidepressant therapy for at least 6 months [15], lack of adherence to pharmacotherapy is unfortunately common in patients with MDDs. In a study in the US on over 520,000 patients using MarketScan databases, adherence to an initial prescription of an antidepressant steadily decreased over a one-year period from 41% at 3 months to 31%, 24%, and 21% at 6, 9, and 12 months, respectively, with similar rates of non-adherence for different classes of antidepressants [16]. In MDD, nonadherence and interruption of the treatment have been linked with several factors, including a poorly perceived safety profile, pharmacological interactions, and low efficacy, with a significant economic burden for both direct and indirect medical costs and lost employment [17].

In Italy, in recent years, several drugs have shown an increase in prescriptions, especially selective serotonin reuptake inhibitors (SSRIs) and serotonin and norepinephrine reuptake inhibitors (SNRIs) [8]. Despite the need for long-term adherence to antidepressant therapy, only about 25-50% of patients with major depression adhere to prescribed treatment [18]. In order to broadly characterize adherence and persistence to therapy in a subset of antidepressants in Italy, we made use of data from the IQVIA [19] Longitudinal Patient Database (LPD). In addition, we have compared data on adherence and persistence with vortioxetine (the last agent marketed and with a different pharmacological and adverse event profile) to other antidepressants.

## 2 MATERIALS AND METHODS

### 2.1 Data Source

This retrospective study used data obtained from the IQVIA LPD, a leading source of medical data in Italy, France, UK, and Belgium, which contains data from general practitioners on drug prescriptions. Data are obtained directly during consultation *via* dedicated management software. The information is gathered in real time and allows patients and physicians to longitudinally monitor prescriptions in a real-life situation. The data collected have given rise to a wide range of studies [20-24]. The present study used data from the Italian database, which involves around 900 GPs and 1,200,000 patients.

### 2.2 Study Population and Design

Selection criteria included patients with at least one prescription for an antidepressant among the 7 most widely prescribed in the IQVIA LPD database in Italy [25] (ATC codes: N06AB04 – citalopram; N06AX21 – duloxetine; N06AB10 – escitalopram; N06AB05 – paroxetine; N06AB06 – sertraline; N06AX16 – venlafaxine; N06AX26 – vortioxetine) during the 2-year period from Apr 1, 2017, to March 31, 2019. The date of the first prescription during the study period was considered the index date. Patients must also have had at least one diagnosis of MDD in the 60 days before or after the index date.

Exclusion criteria included: age <18 and >90 years; at least 1 antidepressant prescription among the entire class of antidepressants (ATC N06A) during a 12-month period before the index date; prescriptions of two or more different antidepressants at the index date; < 1 year of available data after the index date; <1 year of available data before the index date.

### 2.3 Measures of Adherence and Persistence

Adherence was calculated with the Medication Possession Ratio (MPR) method. For each patient, all prescriptions of index date antidepressant molecule starting from the index date (included) to the end of follow-up were collected. Duration of each prescription was calculated based on the defined daily dosage (DDD) (Table **S1**) [26]. The MPR was calculated as the ratio between the total number of days covered by index date antidepressant divided by the total days of follow-up. MPR values higher than 1.0 were truncated at 1.0, according to methodology reported in other studies [16, 27, 28]. For interpretative reasons, MPR was reported in terms of months.

Persistence was defined as the time from initiation to discontinuation of index date antidepressant molecule. Persistence represents the time, measured as days of coverage of the index date antidepressant molecule, from the first to the last prescription. An interruption was defined as an interval of 60 days or more from the last day of coverage of the prescription to the first day of coverage of the following prescription of the same antidepressant molecule, as proposed in other studies [8, 20]. Duration of each prescription was calculated based on DDD (Table **S1**).

Patients were classified according to their level of persistence as continuers: patients who interrupted index date antidepressant treatment for < 60 days during follow-up; intermittent: patients who interrupted index date antidepressant treatment for ≥ 60 days during follow-up and then had a prescription of the same index date antidepressant during follow-up; discontinuers: patients who interrupted index date antidepressant treatment for ≥ 60 days during follow-up and then did not have a prescription of the same index date antidepressant during follow-up.

A sensitivity analysis was carried out using clinical evaluation: from the tables on DDD, some doses are not easily comparable from a clinical standpoint. Given this, starting with dose equivalents from clinical studies with various antidepressants was preferred for a more representative evaluation of the clinical practice (Table **S1**). For calculation of adherence and persistence, the duration of each prescription needs to be considered. Such information was calculated based on the daily dosages equivalent to fluoxetine 40 mg/day, as estimated in another study [29], instead of DDDs. For antidepressants not present in the study, doses equivalent to fluoxetine were calculated through an indirect comparison between the dosage equivalent to fluoxetine [29] and the doses reported in other studies [30-32] (see details in Table **S1**).

### 2.4 Statistical Analysis

Qualitative variables are described using frequencies and percentages. Quantitative variables are described in terms of mean value, standard deviation, median, minimum, and maximum. Chi-square analysis with Bonferroni adjustment was performed to compare frequencies. The key factors associated with low adherence (< 1 month) and high adherence (≥ 6 months) to treatment were identified by Logistic models. A p-value < 0.05 was considered statistically significant. All analyses were performed using SAS^®^ software version 9.4.

## 3 RESULTS

### 3.1 Patient Population and Characteristics

A total of 100,099 patients were identified in the IQVIA LPD database who had at least one prescription for one of the 7 antidepressants during the 2-year study period from 1 April 2017 to 31 March 2019. Most patients (91.8%) were excluded because they were not incident antidepressant users or because a diagnosis of MDD was lacking around the date of the first antidepressant prescription. The final study cohort included 8,235 patients. The study flow is outlined in Fig. (**1**).

Patient demographics and comorbidities are detailed in Table **1**. Overall, the majority of patients were female (67.2%) with age over 65 years (48.1%). The most reported comorbidity was hypertension (45.5%); 46.3% of patients had a history of prior treatment with antidepressants in the 10 years before the index date, while 39.0% were treatment-naive. 14.7% of patients did not have 10 years of available data on IQVIA LPD.

### 3.2 Adherence

Adherence to treatment in the entire group and by antidepressants is shown in Fig. (**2**). Overall, adherence <1 month, ≥1 and <3 months, ≥3 and <6 months was seen in 15.0%, 35.9%, and 16.8% of patients, respectively. In all, 32.4% of the 8,235 patients were adherent to treatment for ≥6 months over a 1-year period. Venlafaxine was associated with the greatest percentage of patients with adherence <1 month (38.3%), and sertraline with the lowest (2.1%). Venlafaxine was associated with the lowest adherence at ≥6 months (21.0%), while sertraline was associated with the highest (40.9%).

Treatment adherence at <1 month and for ≥6 months is shown in Fig. (**3**). The proportion of patients on vortioxetine whose adherence to treatment was <1 month was lower compared to duloxetine (p<0.05), paroxetine (p=NS), and venlafaxine (p<0.05). Considering adherence ≥6 months, the proportion of vortioxetine patients who were adherent ≥6 months was higher than paroxetine (p=NS) and venlafaxine (p<0.05).

Multivariate logistic analysis considering the antidepressant, age, sex, antidepressant treatment history, and comorbidities as covariates was used to estimate predictors of low (<1 month) and high (≥6 months) adherence (Table **2**). In the low adherence model, duloxetine was associated with a 2-times risk of low adherence compared to vortioxetine, while paroxetine was associated with a 34.1% higher risk of low adherence compared to vortioxetine. Venlafaxine was associated with 3-times the risk of low adherence *vs*. vortioxetine, while citalopram, escitalopram, and sertraline were all associated with a significantly lower risk of low adherence compared to vortioxetine (odds ratio (OR) = 0.549, 0.734, and 0.109, respectively). Among the other covariates included in the model, age class (≥65 years) and previous antidepressant treatment were associated with less risk of low adherence. Considering comorbidities, only the presence of baseline coronary artery disease was associated with a higher risk of low adherence.

In the high adherence model, sertraline was associated with a 56.4% higher likelihood of high adherence than vortioxetine, and venlafaxine was associated with a lower likelihood of high adherence compared to vortioxetine (-40.1%). Among the other antidepressants, no other significant differences were seen compared to vortioxetine. Age ≥65 years was associated with a higher likelihood of high adherence, while among comorbidities, the presence of cerebrovascular disease, coronary artery disease, and anxiety were all significantly associated with a higher likelihood of high adherence (all p<0.05).

### 3.3 Persistence

Fig. (**4**) shows treatment persistence over the 12-month period in the entire group and by antidepressants. In all, 68.7% of patients discontinued treatment during follow-up. The highest proportion of patients continuing therapy was seen with duloxetine (23.5%), while the highest proportion of those discontinuing therapy was seen with citalopram (71.3%). No significant differences were seen when comparing the other antidepressants to vortioxetine individually. These results were confirmed in a multivariate logistic model comparing the probability of continuing treatment when adjusted for other covariates (Table **S3**). Only citalopram was associated with a lower likelihood to continue treatment compared to vortioxetine (-25.7%). Women were more likely to continue treatment, as were in general patients aged 41-64 and ≥65 years compared to those aged 18-40. Previous antidepressant therapy was significantly associated with a higher likelihood of not continuing treatment, as was the presence of coronary artery disease at baseline.

### 3.4 Sensitivity Analysis on Adherence and Persistence

Multivariable logistic models were run to estimate predictors of low (<1 month) and high (≥6 months) adherence (calculated based on daily dosages equivalent to fluoxetine 40 mg/day, instead of DDDs), considering the antidepressant, age, sex, antidepressant treatment history, and comorbidities as covariates (Table **3**). Vortioxetine was associated with a lower probability of low adherence compared to all other antidepressants: from 1.8 for sertraline to 5.4 for venlafaxine. In the high adherence analysis, vortioxetine was associated to a higher likelihood of high adherence compared to all other antidepressants (Table **3**).

In a sensitivity analysis of the probability of continuing after the index date treatment, citalopram, escitalopram, paroxetine, and sertraline were associated with a significantly lower probability compared to vortioxetine (OR = 0.480, 0.612, 0.555, and 0.652, respectively), (Table **S4**).

## 4 DISCUSSION

Herein, we used data from the IQVIA LPD to better understand adherence and persistence to therapy in a subset of antidepressants (citalopram, duloxetine, escitalopram, paroxetine, sertraline, venlafaxine) vs. vortioxetine in patients in routine care with GPs in Italy, during a 2-year period from 2017 to 2019. Overall, only about one-third of patients were adherent to therapy for ≥6 months. However, some differences were seen among the different antidepressants: sertraline was associated with an adherence that was roughly twice as high at ≥6 months compared to venlafaxine. Multivariate analysis showed that paroxetine, duloxetine, and venlafaxine were at greater risk of low adherence compared to vortioxetine. Considering the probability of high adherence, sertraline was associated with a higher likelihood than vortioxetine, and venlafaxine was associated with a lower likelihood of high adherence compared to vortioxetine. Also, the comorbidities of the patients influenced adherence to the treatment. In our study, patients with coronary artery disease had a higher risk of low adherence and a lower likelihood of continuing treatment compared to those who did not suffer from this condition.

In terms of treatment persistence, more than two-thirds of patients discontinued therapy during the 12-month period of the study. The greatest percentage of patients continuing therapy was observed with duloxetine, while citalopram was associated with the highest proportion of patients discontinuing therapy.

Lastly, sensitivity analysis, which calculated persistence based on daily dosages equivalent to fluoxetine 40 mg/day instead of DDDs, demonstrated that in this population, the selective serotonin reuptake inhibitors (SSRIs) citalopram, escitalopram, paroxetine, and sertraline were all associated with a significantly lower likelihood of continuing treatment than vortioxetine and a higher risk of low adherence as well. This would suggest that, compared to vortioxetine, SSRIs are associated with poorer persistence and adherence. Interestingly, several other analyses have reported that compared to selective serotonin-norepinephrine reuptake inhibitors (SSNRIs), SSRIs are associated with lower rates of adherence to therapy [16, 33, 34]. Differences in adherence and persistence should be evaluated, keeping in mind the different pharmacodynamic profiles of the drugs. The SSRIs citalopram, escitalopram, paroxetine, sertraline, and vortioxetine all have a presumed serotonergic mechanism of action, while duloxetine and venlafaxine have a serotonergic and noradrenergic mechanism of action [35]. While the primary mechanism of action of SSRIs is similar, each drug has its unique characteristics in terms of pharmacokinetics, pharmacodynamics, and side effects [36]. A meta-analysis of second-generation antidepressants found that the spectrum of adverse events was similar among these agents, although there were some peculiar differences (*e.g.*, higher rates of nausea and vomiting with venlafaxine or increased weight gain with paroxetine) [35]. However, these differences were not associated with any substantial different rates of discontinuation, even if they have the potential to influence the choice of therapy. In addition, it remains unclear if the ability to bind additional 5-HT receptor subtypes, such as in the case of vortioxetine (a multimodal acting antidepressant that functions as a 5-HT3 and 5-HT7 and 5-HT1D receptor antagonist, 5-HT1B receptor partial agonist, 5-HT1A receptor agonist and inhibitor of the 5-HT transporter), brings about any advantages in terms of efficacy or adverse event profile [37]. Better understanding of these implications is further complicated by the possibility that drugs belonging to the same class but targeting more 5-HT receptor subtypes may have different off-target effects and disproportionate risk of dose-related adverse events, which may thus impact adherence to therapy [38]. It has been suggested that the occurrence of side effects with antidepressants, especially in the very early stages of treatment, may be predictive of poor treatment outcomes. This highlights that prescribers should closely monitor patients for adverse reactions to antidepressant therapy [39]. A variety of strategies can be used to manage side effects, including dose reduction, changes to timing, and switching to other agents [40]. Particular attention should be paid to patients with cardiovascular disease, who apparently struggle to maintain adherence to therapy. These individuals should be closely monitored, although no specific cardiovascular side effects are reported, with actually SSRIs being considered protective in terms of cardiovascular risk [41, 42].

The likelihood of remaining adherent during treatment with an antidepressant may depend on the therapeutic class, which underlines the conceivable importance of initial choice of antidepressant therapy. Few studies have reported on rates of adherence to antidepressants considering individual agents. In the above-mentioned study by Sheehan *et al.*, 6-month adherence was 38%, 35%, 34%, 32%, and 31% for venlafaxine extended release, paroxetine-controlled release, escitalopram, duloxetine, and bupropion extended release, respectively. Together with the present results, this shows the concept that adherence to antidepressants may be influenced by the class of drug.

The low overall adherence and persistence to therapy seen with antidepressants herein are largely in line with previous real-world analyses. In 2008, Sheehan *et al.* published the results of a retrospective database analysis of over 250,000 patients [34]. Overall, 33.6% of patients were adherent at 6 months. In 2010, Prukkanone *et al.* reported that 23% of patients with MDD were adherent to therapy over a period of 6 months [43]. On the other hand, Wu *et al.* found that around one-half of patients were adherent to initial antidepressant treatment over 6 months in an analysis limited to SSRIs, serotonin-norepinephrine reuptake inhibitors, or bupropion [44]. In a chart review of 162 patients in 2009, Sawada *et al.* estimated that over 6 months, 56% remained adherent and 44.3% remained persistent to an initial prescription of an antidepressant, irrespective of the pharmacological class [45].

There are several limitations to the present analysis. Firstly, the treatment cohorts and patterns were identified based on claims for a filled prescription, which does not ensure that the patient actually took the medication. In addition, adherence and persistence calculation considered DDD, which may not be representative of actual clinical practice in Italy. For example, it may be that the low DDD for sertraline favored the good adherence and persistence observed herein. One final limitation is that the safety profiles of the individual agents were not considered, and the reasons for discontinuation of therapy are unknown or if discontinuation was a shared decision taken with the prescriber. Vortioxetine is known to have good tolerability, even at high doses [46]. This aspect may have influenced vortioxetine adherence results. In an analysis of older adults, those reporting medication-related side effects were less likely to be adherent to antidepressant therapy [47]. Another limitation is the absence of data regarding other antidepressants belonging to other classes, such as tricyclics, trazodone, bupropion, agomelatine, and ketamine/esketamine. However, their use nowadays in Italy is usually dedicated to specific patient populations or treatment resistant cases [48-51].

Achieving optimal therapy with antidepressants requires an adequate duration of treatment and tailoring the dose to treat the full range of symptoms while minimizing adverse events [52]. The need to continue therapy with an antidepressant was highlighted by a recent randomized trial in primary care. In patients who felt well enough to discontinue treatment, those discontinuing their antidepressants had a higher risk of relapse of depression at one year compared to patients who continued antidepressant treatment [53]. In considering other means of optimizing adherence, a recent study investigating the use of a personal support program (tAccess Patient Support Program) for patients prescribed vortioxetine therapy reported adherence of 83% and 70% at 84 and 180 days, respectively, suggesting that the intervention may help to increase adherence and help ensure that patients complete their planned therapy [54]. The present study further strengthens the need to carry out additional research on antidepressants and identify clinical and other factors that influence adherence and persistence to therapy. Lastly, adherence may depend on the therapeutic class. In this analysis of a GP prescriptions database in patients in Italy, vortioxetine adherence and persistence at one year appeared to be in line with the other antidepressant treatments, even if overall adherence was relatively low.

## CONCLUSION

Analysis of the IQVIA LPD revealed how adherence and persistence to antidepressant treatment vary according to age, previous drug therapy, and comorbidities. Overall, one-third of patients with MDDs were adherent to antidepressant therapy for ≥6 months over a 1-year period. Compared to vortioxetine, patients treated with duloxetine, paroxetine and venlafaxine were significantly associated with a higher likelihood of low adherence. These results seem strengthened by the sensitivity analysis carried out for a more representative evaluation of the clinical practice, where vortioxetine was significantly associated with a lower risk of low adherence compared to all other antidepressants.

Overall, persistence to therapy was low, with 68.7% of patients discontinuing treatment during follow-up. Among the 7 drugs analyzed, the highest percentage of patients continuing therapy was seen with duloxetine, while citalopram was associated with the highest proportion of patients discontinuing therapy. Compared to the overall cohort of patients, the proportion of patients continuing therapy was slightly higher with vortioxetine, even if no significant differences were observed when comparing vortioxetine to the other antidepressants. Previous antidepressant therapy was associated with a higher likelihood of not continuing treatment as well as coronary artery disease at baseline. Considering the analysis based on the equivalent doses to fluoxetine, vortioxetine was significantly associated with a higher likelihood of continuing treatment compared to citalopram, escitalopram, paroxetine, and sertraline.

## Figures and Tables

**Fig. (1):**
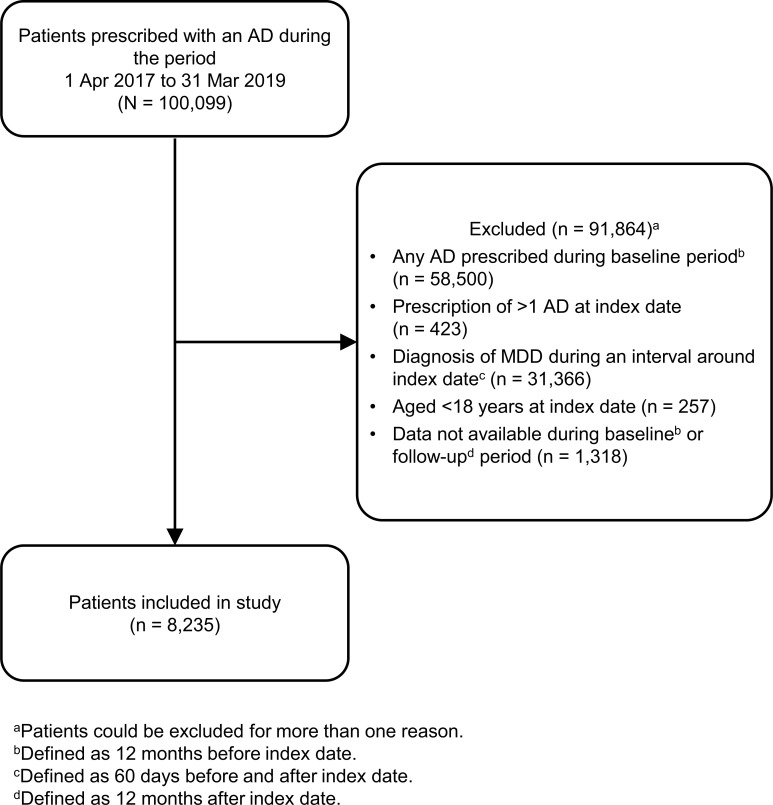
Patient flow diagram.

**Fig. (2):**
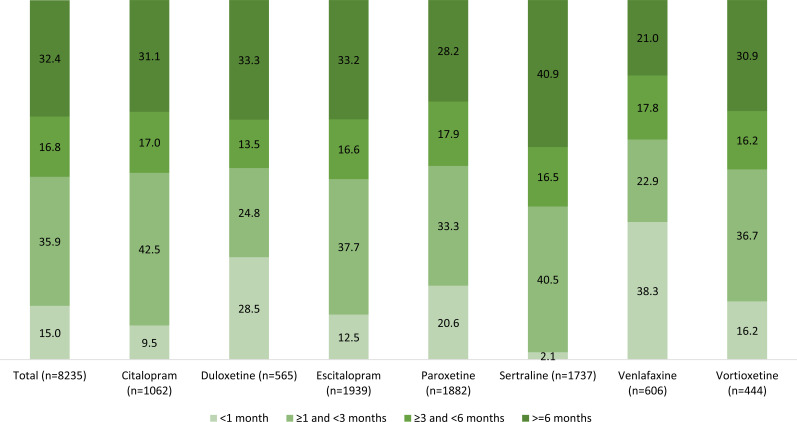
Treatment adherence measured in the 12-month follow-up period after index date and stratified by antidepressant.

**Fig. (3):**
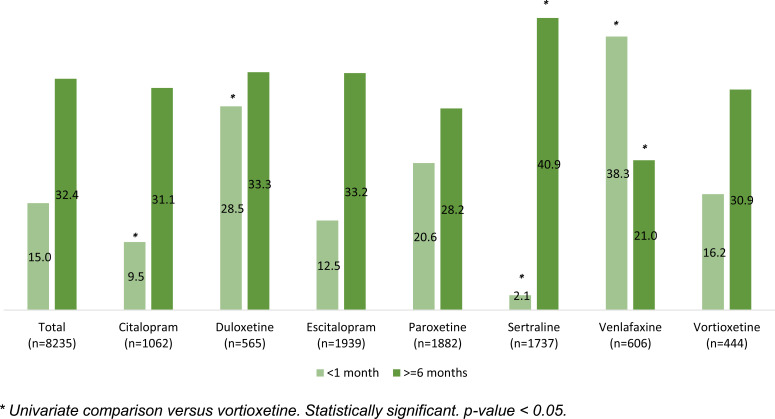
Treatment adherence for patients in treatment with vortioxetine and other antidepressants.

**Fig. (4):**
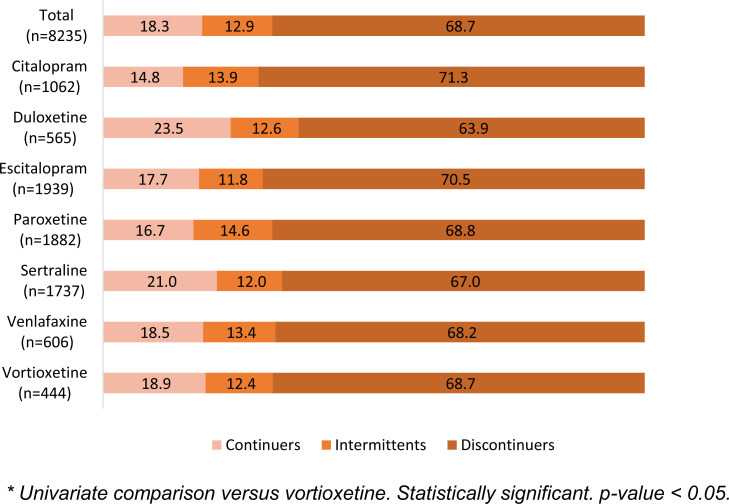
Treatment persistence measured in the 12-month follow-up period after the index date and stratified by antidepressant.

**Table 1:** Patient demographic characteristics and comorbidities, stratified by antidepressant.

-	-	**OVERALL**		**Antidepressant Molecule**													
				**Citalopram**		**Duloxetine**		**Escitalopram**		**Paroxetine**		**Sertraline**		**Venlafaxine**		**Vortioxetine**	
-	N (%)	8235	-	1062	-	565	-	1939	-	1882	-	1737	-	606	-	444	-
**Gender**	-	-	-	-	-	-	-	-	-	-	-	-	-	-	-	-	-
Female	n (%)	5535	67.2	744	70.1	389	68.9	1324	68.3	1285	68.3	1122	64.6	395	65.2	276	62.2
**Age^a^**	-	-	-	-	-	-	-	-	-	-	-	-	-	-	-	-	-
≤40 years old	n (%)	994	12.1	103	9.7	46	8.1	254	13.1	234	12.4	204	11.7	85	14.0	68	15.3
41-64 years old	n (%)	3279	39.8	391	36.8	249	44.1	798	41.2	810	43.0	588	33.9	269	44.4	174	39.2
≥65 years old	n (%)	3962	48.1	568	53.5	270	47.8	887	45.8	838	44.5	945	54.4	252	41.6	202	45.5
**Comorbidities^b^**	-	-	-	-	-	-	-	-	-	-	-	-	-	-	-	-	-
Hypertension	n (%)	3838	45.5	521	44.9	274	43.6	894	47.2	796	46.5	872	44.7	273	44.8	208	43.6
Hyperlipidemia	n (%)	1804	21.4	241	20.8	144	22.9	400	21.1	409	23.9	391	20.1	122	20.0	97	20.3
Diabetes	n (%)	1145	13.6	158	13.6	92	14.6	242	12.8	225	13.1	274	14.1	89	14.6	65	13.6
Cerebrovascular Disease	n (%)	969	11.5	141	12.2	77	12.2	193	10.2	169	9.9	240	12.3	76	12.5	73	15.3
Coronary Artery Disease	n (%)	674	8.0	99	8.5	42	6.7	164	8.7	113	6.6	172	8.8	50	8.2	34	7.1
Anxiety	n (%)	876	9.1	117	9.4	60	9.0	200	8.9	228	10.2	180	8.9	55	7.7	36	6.9
**Previous history of AD treatment**	-	-	-	-	-	-	-	-	-	-	-	-	-	-	-	-	-
Unknown^c^	n (%)	1212	14.7	148	13.9	72	12.7	288	14.9	279	14.8	247	14.2	97	16.0	81	18.2
Naive	n (%)	3209	39.0	405	38.1	215	38.1	771	39.8	717	38.1	714	41.1	205	33.8	182	41.0
Non-naive	n (%)	3814	46.3	509	47.9	278	49.2	880	45.4	886	47.1	776	44.7	304	50.2	181	40.8

^a^ age calculated at index date; ^b^ comorbidities 1- year pre index date; ^c^ patients without 10 years of available data in IQVIA LPD.

**Table 2:** Multivariable logistic model of the risk of low (<1 month) and high adherence (≥6 months); DDD analysis.

	**Low Adherence (< 1 Month)**		**High Adherence (≥ 6 Months)**	
**Parameter**	**OR (95% CI)**	***p*-value^a^**	**OR (95% CI)**	***p*-value^a^**
Antidepressant	-	-	-	-
Vortioxetine (**REF**)	-	-	-	-
Citalopram	0.549 (0.396-0.760)	**0.0003**	1.01 (0.794-1.285)	0.9347
Duloxetine	2.099 (1.535-2.871)	**<.0001**	1.119 (0.856-1.462)	0.4127
Escitalopram	0.734 (0.551-0.977)	**0.0344**	1.126 (0.900-1.408)	0.2994
Paroxetine	1.341 (1.016-1.769)	**0.0384**	0.886 (0.706-1.11)	0.2927
Sertraline	0.109 (0.072-0.166)	**<0.0001**	1.564 (1.250-1.957)	**<0.0001**
Venlafaxine	3.248 (2.401-4.393)	**<0.0001**	0.599 (0.452-0.794)	**0.0004**
Sex	-	-	-	-
Male (**REF**)	-	-	-	-
Female	1.01 (0.881-1.159)	0.8850	1.079 (0.975-1.194)	0.1424
Age group category, years	-	-	-	-
18-40 (**REF**)	-	-	-	-
41-64	0.896 (0.730-1.101)	0.2972	1.159 (0.986-1.361)	0.0735
≥65	0.797 (0.636-0.999)	**0.0485**	1.191 (1.002-1.415)	**0.0478**
Previous AD treatment 10 years before index date	-	-	-	-
No (**REF**)	-	-	-	-
Unknown	0.817 (0.672-0.993)	**0.0423**	1.326 (1.151-1.527)	**<0.0001**
Yes	0.787 (0.686-0.903)	**0.0006**	0.991 (0.894-1.097)	0.8573
Comorbidity	-	-	-	-
Hypertension	1.006 (0.870-1.163)	0.9359	1.053 (0.947-1.171)	0.3407
Diabetes (type 1 or 2)	0.925 (0.758-1.128)	0.4422	0.968 (0.841-1.116)	0.6577
Coronary Artery Disease	1.389 (1.096-1.761)	**0.0065**	0.796 (0.664-0.954)	**0.0134**
Cerebrovascular Disease	0.862 (0.692-1.073)	0.1826	1.196 (1.031-1.387)	**0.0182**
Anxiety	1.068 (0.872-1.307)	0.5253	1.201 (1.035-1.393)	**0.0155**

^a^ Values in bold indicate parameters with significantly increased risk of low adherence/likelihood of high adherence.

CI, confidence interval; OR, odds ratio; REF, reference.

**Table 3:** Multivariable logistic model of the risk of low adherence (< 1 month)/likelihood of high adherence (≥6 months) – sensitivity analysis.

	**Low Adherence (< 1 Month)**		**High Adherence (≥ 6 Months)**	
**Parameter**	**OR (95% CI)**	***p*-value^a^**	**OR (95% CI)**	***p*-value^a^**
**Antidepressant**	-	-	-	
**Vortioxetine (REF)**	-	-	-	
Citalopram	2.421 (1.821-3.218)	**<.0001**	0.416 (0.320-0.541)	**<.0001**
Duloxetine	2.612 (1.919-3.554)	**<.0001**	0.57 (0.427-0.76)	**0.0001**
Escitalopram	2.088 (1.591-2.739)	**<.0001**	0.469 (0.371-0.593)	**<.0001**
Paroxetine	2.485 (1.895-3.259)	**<.0001**	0.38 (0.299-0.483)	**<.0001**
Sertraline	1.843 (1.400-2.425)	**<.0001**	0.526 (0.416-0.666)	**<.0001**
Venlafaxine	5.374 (3.984-7.249)	**<.0001**	0.262 (0.188-0.364)	**<.0001**
Sex	-	-	-	-
Male (**REF**)	-	-	-	-
Female	1.086 (0.980-1.205)	0.1161	1.119 (0.986-1.27)	0.0811
Age group category, years	-	-	-	-
18–40 (**REF**)	-	-	-	-
41-64	0.984 (0.839-1.154)	0.8422	1.236 (1.011-1.511)	**0.0389**
≥65	0.925 (0.778-1.099)	0.3765	1.08 (0.869-1.341)	0.4885
Previous AD treatment 10 years before index date	-	-	-	-
No (**REF**)	-	-	-	-
Unknown	0.825 (0.713-0.955)	**0.0101**	1.131 (0.953-1.343)	0.1589
Yes	0.758 (0.684-0.841)	**<.0001**	0.86 (0.758-0.977)	**0.0201**
Comorbidity	-	-	-	-
Hypertension	0.98 (0.879-1.092)	0.7097	1.069 (0.937-1.219)	0.3219
Diabetes (type 1 or 2)	0.95 (0.821-1.099)	0.4892	0.993 (0.832-1.184)	0.9361
Coronary Artery Disease	1.328 (1.113-1.583)	**0.0016**	0.77 (0.609-0.973)	**0.0287**
Cerebrovascular Disease	0.894 (0.763-1.047)	0.1643	1.209 (1.009-1.449)	**0.0400**
Anxiety	0.969 (0.830-1.131)	0.6889	1.26 (1.054-1.505)	**0.0110**

^a^Values in bold indicate parameters with significantly increased risk of low adherence/likelihood of high adherence.

CI, confidence interval; OR, odds ratio; REF, reference.

## Data Availability

The data supporting the findings are available from IP and RC upon reasonable request and with permission of IQVIA. Restrictions may be applied to their availability. Data were used under license for the current analysis and are not publicly available.

## LIST OF ABBREVIATIONS

DDD: Defined Daily Dosage
LPD: Longitudinal Patient Database
MDDs: Major Depressive Disorders
SNRIs: Serotonin and Norepinephrine Reuptake Inhibitors
SSRIs: Selective Serotonin Reuptake Inhibitors
