# Supplementary material for: Adherence to, and Persistence of, Antidepressant Therapy in Patients with Major Depressive Disorder: Results from a Population-based Study in Italy
Source: Curr Neuropharmacol. 2023 Mar 8;21(3):727–39. doi: 10.2174/1570159X20666220411092813 (PMC10207922; doi:10.2174/1570159X20666220411092813)
Supplement: Supplementary file 1 [file CN-21-727_SD1.pdf]

Supplementary Material

Adherence to, and Persistence of, Antidepressant Therapy in Patients with Major Depressive Disorder: Results from a Population-based Study in Italy

Marco Di Nicola[1], Bernardo Dell'Osso[2], Ilaria Peduto[3], Riccardo Cipelli[3], Anna Cristina Pugliese[4], Maria Salvina Signorelli[5], Antonio Ventriglio[6] and Giovanni Martinotti[7,8,9,*]

[1]Department of Psychiatry, Fondazione Policlinico Universitario Agostino Gemelli IRCCS, Rome, Italy; Department of Neuroscience, Section of Psychiatry, Università Cattolica del Sacro Cuore, Rome, Italy; [2]Department of Biomedical and Clinical Sciences Luigi Sacco, Aldo Ravelli Center for Neurotechnology and Brain Therapeutic, University of Milan, Milan, Italy; [3]IQVIA Solutions Srl, Milano, Italy; [4]Medical Department Lundbeck, Italia; [5]Department of Clinical and Experimental Medicine, Psychiatry Unit, University of Catania, via Santa Sofia 78, 95123 Catania, Italy; [6]Department of Clinical and Experimental Medicine, University of Foggia, Foggia, Italy; [7]Department of Neuroscience, Imaging and Clinical Sciences, University "G. D'Annunzio" of Chieti - Pescara, Chieti, Italy; [8]Psychopharmacology, Drug Misuse & Novel Psychoactive Substances Research Unit, School of Life and Medical Sciences, University of Hertfordshire, Hertfordshire, UK; [9]SRP "Villa Maria Pia", Rome, Italy

Table 1S. DDDs and daily dosages equivalent to fluoxetine 40 mg/day for each antidepressant.

Molecule	DDD (mg/day)	Daily Dosages Equivalent to Fluoxetine 40mg/d	References
CITALOPRAM	20	36.5	[1, 2]
DULOXETINE	60	83.1	[1, 3]
ESCITALOPRAM	10	18	[1]
PAROXETINE	20	34	[1]
SERTRALINE	50	98.5	[1]
VENLAFAXINE	100	149.4	[1]
VORTIOXETINE	10	10	[1, 4]

Table 2S. List of ICD-9-CM codes used in the study.

Diagnosis	ICD-9-CM
MDD	296.2 (Major depressive disorder single episode)<br>296.3 (Major depressive disorder recurrent episode)<br>311.x (Depressive disorder, not elsewhere classified)
Hyperlipidemia	272.0x (Pure hypercholesterolemia)<br>272.2x (Mixed hyperlipidemia)<br>272.4x (Other and unspecified hyperlipidemia)
Diabetes	250.xx (Diabetes Mellitus)
Hypertension	401.xx (Essential Hypertension)
Coronary Artery Disease	410.xx (Acute myocardial infarction)<br>411.xx (Other acute and subacute forms of ischemic heart disease)<br>412.xx (Old myocardial infarction)<br>413.xx (Angina pectoris)<br>414.xx (Other forms of chronic ischemic heart disease)
Cerebrovascular Disease	433.xx (Occlusion and stenosis of precerebral arteries)<br>434.xx (Occlusion of cerebral arteries)<br>435.xx (Transient cerebral ischemia)<br>436.xx (Acute, but ill-defined, cerebrovascular disease)<br>437.xx (Other and ill-defined cerebrovascular disease)<br>438.xx (Late effects of cerebrovascular disease)

Table 3S. Multivariable logistic model of the likelihood to continue the index date treatment – DDD analysis.

**Parameter**	**OR (95% CI)**	***p*-value[a]**
Antidepressant		
Vortioxetine (**REF**)		
Citalopram	0.743 (0.554–0.995)	**0.0466**
Duloxetine	1.310 (0.962–1.782)	0.0863
Escitalopram	0.931 (0.714–1.215)	0.5995
Paroxetine	0.864 (0.661–1.129)	0.2837
Sertraline	1.152 (0.884–1.502)	0.2941
Venlafaxine	0.984 (0.718–1.348)	0.9193
Sex		
Male (**REF**)		
Female	1.174 (1.038–1.329)	**0.0107**
Age group category, years		
18–40 (**REF**)		
41-64	1.376 (1.125–1.684)	**0.0019**
≥65	1.256 (1.011–1.559)	**0.0391**
Previous AD treatment 10 years before index date		
No (**REF**)		
Unknown	1.125 (0.951–1.331)	0.1680
Yes	0.861 (0.761–0.973)	**0.0168**
Comorbidity		
Hypertension	1.097 (0.966–1.246)	0.1530
Diabetes (type 1 or 2)	1.010 (0.853–1.197)	0.9049
Coronary Artery Disease	0.786 (0.628–0.984)	**0.0358**
Cerebrovascular Disease	1.164 (0.976–1.389)	0.0902
Anxiety	1.097 (0.966–1.246)	0.1530

[a] Values in bold indicate parameters with significantly increased likelihood of continue the treatment.
CI, confidence interval; OR, odds ratio; REF, reference.

Table 4S. Multivariable logistic model of the likelihood of continue the index date treatment – sensitivity analysis.

Parameter	OR (95% CI)	*p*-value[a]
Antidepressant		
Vortioxetine (**REF**)		
Citalopram	0.480 (0.351–0.655)	**<.0001**
Duloxetine	1.115 (0.814–1.526)	0.4977
Escitalopram	0.612 (0.465–0.805)	**0.0005**
Paroxetine	0.555 (0.420–0.734)	**<.0001**
Sertraline	0.652 (0.494–0.860)	**0.0025**
Venlafaxine	0.868 (0.630–1.197)	0.3885
Sex		
Male (**REF**)		
Female	1.246 (1.081–1.437)	**0.0024**
Age group category, years		
18–40 (**REF**)		
41-64	1.409 (1.117–1.777)	**0.0038**
≥65	1.217 (0.948–1.562)	0.1238
Previous AD treatment 10 years before index date		
No (**REF**)		
Unknown	1.055 (0.873–1.276)	0.5784
Yes	0.772 (0.671–0.888)	**0.0003**
Comorbidity		
Hypertension	1.121 (0.969–1.296)	0.1233
Diabetes (type 1 or 2)	1.037 (0.856–1.256)	0.7079
Coronary Artery Disease	0.877 (0.682–1.128)	0.3074
Cerebrovascular Disease	1.148 (0.940–1.402)	0.1759
Anxiety	1.292 (1.064–1.569)	**0.0098**

[a] Values in bold indicate parameters with significantly increased likelihood of continue the treatment.
CI, confidence interval; OR, odds ratio; REF, reference.

REFERENCES

[1] Hayasaka, Y.; Purgato, M.; Magni, L.R.; Ogawa, Y.; Takeshima, N.; Cipriani, A.; Barbui, C.; Leucht, S.; Furukawa, T.A. Dose equivalents of antidepressants: Evidence-based recommendations from randomized controlled trials. *J Affect Disord*. **2015**; *180* 179-84. 10.1016/j.jad.2015.03.021

[2] Lepola, U.M.; Loft, H.; Reines, E.H. Escitalopram (10-20 mg/day) is effective and well tolerated in a placebo-controlled study in depression in primary care. *Int Clin Psychopharmacol*. **2003**; *18*(4), 211-7. 10.1097/00004850-200307000-00003

[3] Khan, A.; Bose, A.; Alexopoulos, G.S.; Gommoll, C.; Li, D.; Gandhi, C. Double-blind comparison of escitalopram and duloxetine in the acute treatment of major depressive disorder. *Clin Drug Investig*. **2007**; *27*(7), 481-92. 10.2165/00044011-200727070-00005

[4] Wang, G.; Gislum, M.; Filippov, G.; Montgomery, S. Comparison of vortioxetine versus venlafaxine XR in adults in Asia with major depressive disorder: a randomized, double-blind study. *Curr Med Res Opin*. **2015**; *31*(4), 785-94. 10.1185/03007995.2015.1014028
